# Author’s response

**DOI:** 10.52054/FVVO.16.3.043

**Published:** 2024-09-30

**Authors:** G Hudelist, B Gerges, G Condous

Dear Editor,

We would like to thank Koninckx et al. (2024) for their critical comments. In response to the points raised by Koninckx and colleagues we would like to make some clarifying responses. The authors of this letter mention “…the overall poor methodological quality of these studies” and that in the evidence included “…the preva- lence of deep endometriosis was not considered”. We would like to remind Koninckx et al. that the work is a consensus statement but neither a systematic review on the accuracy of ultrasound for detecting endometriosis nor an original work on the test accuracy of transvaginal sonography (Condous et al., 2024). The comments of the author are correct in the way that the grade of evidence we have derives from studies of both lower and higher quality. This is, by the way, a problem that evidence-based medicine is confronted with. Consensus derives from the Latin word consentio and means “feel together” or “agree”.

The consensus statement published is the result of such a process. This process of “agreeing” was based on several factors; the current evidence, the opinion of the authors and – of course – their personal experience. Opinions are based on these issues but should not solely be corrected for experience as suggested by Koninckx et al. (2024). Finally, the manuscript of course underwent peer-review. To our surprise, one co-author of this letter took part in the consensus process as well. Whether a change of opinion or some kind of misunderstand- ing of the very final version of the consensus work may explain this, remains unknown.
